# Professor White and This Issue of the Journal

**Published:** 1881-11

**Authors:** 


### Professor White and this Issue of the Journal.

So much has been already said and written in this vicinity concerning the late Professor White that any further encomiums coming so tardily, seem almost inappropriate and ill-advised. Nor do high-sounding eulogies in any case alter the estimation of a man's character in the minds of those among whom he has lived. We write our epitaphs with our own actions. It is therefore superfluous to indulge in glittering generalities concerning the life of one so well known as Dr. White, although a word of introduction to the following biographical sketch seems eminently proper.

This sketch is given at some length as the professional record of a man whose well marked characteristics have made him a historical personage in the medical annals of this vicinity.

As a teacher he came in contact with medical men, not by hundreds, but actually by thousands; as a writer he was a contributor to some of the best journals in the country; and as an operator in gynecology he possessed an experience seldom equalled. It is not suprising, therefore, that his death should make a profound impression upon those with whom he lived. Moreover, as this journal has a large circulation among his friends and among the alumni of the Medical Department of the University of Buffalo, it seemed advisable to devote a considerable part of the present number to a consideration of his character and works.

We offer no further apology, therefore, for reproducing portions of articles which have already been published by him, believing that they will prove acceptable from their intrinsic worth aside from the personal interest attached to them. The steel engraving frontispiece was copied from a picture taken some few years ago, but may still be considered a good likeness. The biographical sketch necessarily contains many data which have heretofore been publicly noticed, but his career is here treated, especially, from a medical standpoint, and as such, is the record of an active life spent in advancing his profession and in the services of humanity.

